# Fibromatosis Colli: A case report

**DOI:** 10.1016/j.radcr.2021.12.009

**Published:** 2021-12-28

**Authors:** Siham Nasri, Ihssane Afilal, Zakariae Missaoui, Hanane El Aggari, Imane Kamaoui, Narjiss Aichouni, Imane Skiker

**Affiliations:** Department of Radiology, Mohammed VI University Hospital, Faculty of Medicine, University Mohammed First, Oujda, Morocco

**Keywords:** Fibromatosis Colli, Pseudotumor, Sternocleidomastoid Muscle, Ultrasound, MRI

## Abstract

Fibromatosis Colli also known as congenital torticollis is a rare cause of benign cervical pseudotumor in neonates, consisting of benign fibrous tissue proliferation within the sternocleidomastoid muscle, resulting in a fusiform enlargement. The cause of fibrosis is unknown; however it could be linked to trauma during pregnancy or at the time of birth, resulting in hemorrhage and, subsequently, fibrosis. Ultrasound plays a necessary role in confirming this diagnosis and follow-up. We Report a case of Fibromatosis colli in a four-week old newborn who was diagnosed using ultrasonography and treated with physiotherapy.

## Introduction

Fibromatosis Colli is a rare condition of benign cervical masse seen in newborns. It is a focal or diffuse enlargement of the sternocleidomastoid muscle giving an aspect of a cervical pseudotumor. Though the exact etiology is still unknown, it has been reported that birth trauma or muscle injury in utero could be incriminated in the appearance of this pseudotumor. The diagnosis is mainly clinical, the unilateral cervical enlargement could be associated with facial asymmetry and limited movement of the neck, called congenital torticollis. However, the ultrasound is able to confirm the diagnosis, eliminate the differentials and ensure the follow-up. This pseudotumor natural evolution is usually spontaneous in 4 to 8 months requiring only physiotherapy as treatment. We present a case report of congenital torticollis in a four-week-old newborn who had a right cervical swelling that the mother had noticed a few days before.

## Observation

**Fig. 1:**
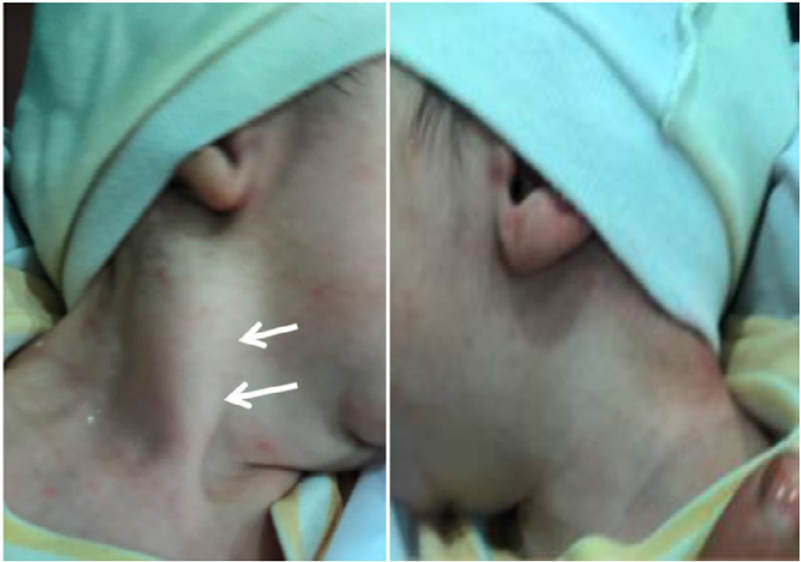
Right lateral cervical tumefaction (white arrow) with no inflammatory signs compared to the left normal side.

A four-week-old newborn (male), was admitted with right cervical swelling and restricted neck movement. The obstetrical history of the mother revealed that the pregnancy reached full term, but was poorly monitored, and the newborn was delivered by a natural assisted birth, with dystocia due to breech presentation. The clinical examination revealed a swelling of firm consistency without any inflammatory sings Fig. 1. No other symptom was associated.

**Fig. 2:**
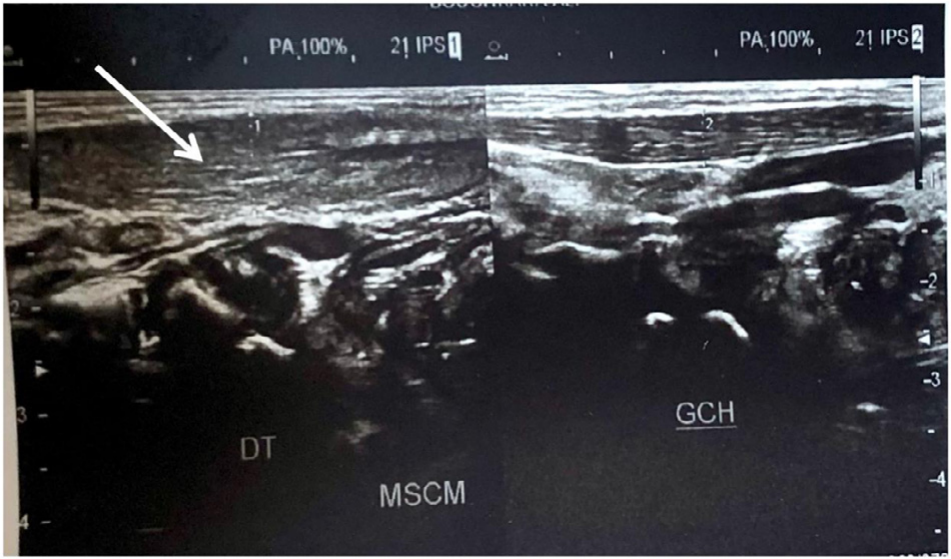
Ultrasound appearance of homogeneous fusiform hypertrophy of the right SCM muscle (white arrow) compared to the left SCM muscle.

**Fig. 3:**
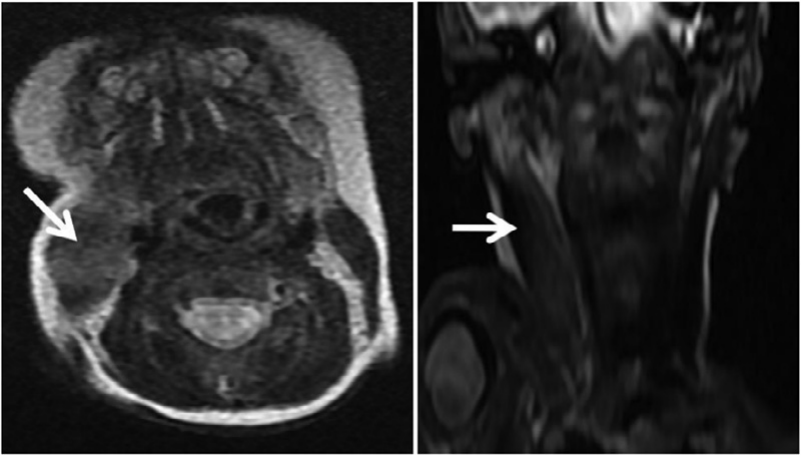
MRI T2 axial and coronal sequence showing the fusiform hypertrophy of the right sternocleidomastoid muscle in hypersignal (white arrow) compared to the left one.

A cervical ultrasound revealed a fusiform thickening of the right sternocleidomastoid muscle, which was homogeneous in echo-structure, with a preserved muscle's fibrillar structure. No significant change in internal vascularity was seen. This pseudotumor measured 9.3 mm compared to 4.9 mm on the left side, without mass effect on the vascular axis or the adjacent structures Fig. 2. There were associated cervical lymphadenomegalies. Otherwise, the rest of the cervical ultrasound was normal. Based on these findings, we were able to confirm the diagnosis of right sided Fibromatosis Colli. An MRI was preformed to confirm the diagnosis and eliminate any differentials. It showed the fusiform pseudotumor of the right sternocleidomastoid muscle on T2 sequence with increased signal intensity compared to the contralateral muscle Fig. 3.

The newborn benefited from motor physiotherapy, the physician advised his parents to gently rotate the head of the child toward the side of the lesion, five -to six times daily and hold it for a few seconds.

## Discussion

Fibromatosis Colli is a rare condition, with a prevalence estimated at 0.4%. It is categorized as a benign fibroblastic proliferation of the sternocleidomastoid muscle [1]. Newborns are usually normal at birth and present with a unilateral neck mass associated to restricted motion, occurring two to four weeks after birth. It is located on the right side in 73 % of cases, and mainly concerns the male gender [2]. This condition is usually associated to birth trauma and hard labor such as forceps use or breech delivery, even if the exact physiopathology is still a subject of controversy. In fact, some cases of Fibromatosis Colli were reported without any history of birth trauma. The etiologies of Fibromatosis Colli could be linked to a fetal malposition in-utero, trauma to the muscle or decrease in blood flow during pregnancy or during delivery, eventually leading to muscle fibrosis. Other causes have been reported like infection or heredity. The infectious hypothesis could be explained by a septic thrombus that reduces blood flow to the sternocleidomastoid muscle. Heredity however, is evoked in familial congenital torticollis reported in siblings, especially if there is no notion of birth trauma.

Fibromatosis Colli is, first of all, a clinical diagnosis and is suspected in cases of unilateral cervical swelling and history of birth trauma. However, ultrasound is the key imaging modality to confirm the diagnosis, with a sensitivity of 100%, it is also able to eliminate adenopathies or invasion and mass effect on the adjacent structures and ensures the follow-up. It is a gold standard in case of Fibromatosis Colli, due to its availability, low cost and absence of irradiation. It typically shows a fusiform or an ellipsoid shaped thickening of the sternocleidomastoid muscle [3]. Margins are usually well defined. The presence of hyperechoic foci in its center could be correlated to a previous hemorrhaging, but a mass or collection should not be individualized.

In case the ultrasound is inconclusive, we can further assess the pseudotumor using CT, although this exposes the newborn to ionizing radiation, and it shows an iso-attenuated enlargement of the sternocleidomastoid muscle with normal surrounding structures [4]. MRI shows a hyperintense homogenous pseudotumor of this muscle on T2-weighted sequences, compared to normal muscle. This imaging modality is also helpful in confirming the absence of any airway or vascular compression, thus eliminating other differentials. Biopsy of the mass in not advised, however, cytology shows bland appearing fibroblasts with degenerate and atrophic smooth muscle and no evidence of hemorrhage or inflammation. Collagen is seen along with a number of bland, bare nuclei and muscle giant cells in the background [5]. Treatment is mainly conservative; in fact, most reports have applied the conservative method consisting of observation and physiotherapy by gently and repeatedly turning the newborn's neck towards the side of the lesion five to six times a day and holding it in that position for a few seconds [6], over a period of a few months, until the swelling begins to subside. Even without any treatment, Fibromatosis Colli tends to spontaneously subside in four to eight months. However, Botulinum toxin type A injections or surgical tenomyotomy, can be considered as alternative treatments in refractory cases (<10%) [7].

## Conclusion

Fibromatosis Colli is a rare benign pseudotumor in neonates. The diagnosis is mainly clinical and can be confirmed using ultrasonography that is an ideal, easily available, low- cost imaging modality that also ensures the follow-up. MRI can be useful in doubtful cases. The evolution is usually gradual and spontaneous and can be accelerated using simple physiotherapy techniques.

## Informed consent

An informed consent was obtained from the patient.
